# Pre-treatment With Fasudil Prevents Neomycin-Induced Hair Cell Damage by Reducing the Accumulation of Reactive Oxygen Species

**DOI:** 10.3389/fnmol.2019.00264

**Published:** 2019-11-06

**Authors:** Yanqiu Zhang, Wei Li, Zuhong He, Yunfeng Wang, Buwei Shao, Cheng Cheng, Shasha Zhang, Mingliang Tang, Xiaoyun Qian, Weijia Kong, Hui Wang, Renjie Chai, Xia Gao

**Affiliations:** ^1^Jiangsu Provincial Key Medical Discipline (Laboratory), Department of Otolaryngology Head and Neck Surgery, Nanjing Drum Tower Hospital Clinical College of Nanjing Medical University, Nanjing, China; ^2^MOE Key Laboratory for Developmental Genes and Human Disease, Institute of Life Sciences, Jiangsu Province High-Tech Key Laboratory for Bio-Medical Research, Southeast University, Nanjing, China; ^3^Research Institute of Otolaryngology, Nanjing, China; ^4^Department of Otolaryngology Head and Neck Surgery, Xuzhou Cancer Hospital, Xuzhou, China; ^5^Department of Otolaryngology Head and Neck Surgery, The Affiliated Hospital of Xuzhou Medical University, Xuzhou, China; ^6^Department of Otorhinolaryngology, Union Hospital, Tongji Medical College, Huazhong University of Science and Technology, Wuhan, China; ^7^Key Laboratory of Hearing Medicine of NHFPC, State Key Laboratory of Medical Neurobiology, ENT Institute and Otorhinolaryngology Department of Affiliated Eye and ENT Hospital, Shanghai Engineering Research Centre of Cochlear Implant, Fudan University, Shanghai, China; ^8^Shanghai Fenyang Vision & Audition Center, Shanghai, China; ^9^Department of Otolaryngology Head and Neck Surgery, Shanghai Jiao Tong University Affiliated Sixth People’s Hospital, Shanghai, China; ^10^Co-Innovation Center of Neuroregeneration, Nantong University, Nantong, China; ^11^Institute for Stem Cell and Regeneration, Chinese Academy of Science, Beijing, China; ^12^Beijing Key Laboratory of Neural Regeneration and Repair, Capital Medical University, Beijing, China

**Keywords:** ototoxic, hair cell, fasudil, aminoglycoside, reactive oxygen species, rho signaling pathway

## Abstract

Ototoxic drug-induced hair cell (HC) damage is one of the main causes of sensorineural hearing loss, which is one of the most common sensory disorders in humans. Aminoglycoside antibiotics are common ototoxic drugs, and these can cause the accumulation of intracellular oxygen free radicals and lead to apoptosis in HCs. Fasudil is a Rho kinase inhibitor and vasodilator that has been widely used in the clinic and has been shown to have neuroprotective effects. However, the possible application of fasudil in protecting against aminoglycoside-induced HC loss and hearing loss has not been investigated. In this study, we investigated the ability of fasudil to protect against neomycin-induced HC loss both *in vitro* and *in vivo*. We found that fasudil significantly reduced the HC loss in cochlear whole-organ explant cultures and reduced the cell death of auditory HEI-OC1 cells after neomycin exposure *in vitro*. Moreover, we found that fasudil significantly prevented the HC loss and hearing loss of mice in the *in vivo* neomycin damage model. Furthermore, we found that fasudil could significantly inhibit the Rho signaling pathway in the auditory HEI-OC1 cells after neomycin exposure, thus further reducing the neomycin-induced accumulation of reactive oxygen species and subsequent apoptosis in HEI-OC1 cells. This study suggests that fasudil might contribute to the increased viability of HCs after neomycin exposure by inhibition of the Rho signaling pathway and suggests a new therapeutic target for the prevention of aminoglycoside-induced HC loss and hearing loss.

## Introduction

Hearing loss is the most common sensory deficiency worldwide, and the number of people suffering from hearing loss is rising due to aging populations, ototoxic drug abuse, noise exposure, and environmental pollution. Ototoxic drugs (including many chemotherapeutics, loop diuretics, and aminoglycosides) induce hair cell (HC) damage and are the one of the common cause of sensorineural hearing loss (Tabuchi et al., 2011). Aminoglycoside antibiotics are widely used due to their low cost and effectiveness against serious infections caused by gram-negative bacteria, but these drugs can cause HC death by activating apoptosis and necrosis-related pathways (Sugahara et al., 2006; Tabuchi et al., 2011). Thus, how to reduce the ototoxicity of aminoglycosides is a primary focus in the hearing research field. The outer HCs are the most vulnerable targets for aminoglycosides, but as the dose or the duration of aminoglycoside treatment increases, the inner HCs are also damaged (Karasawa et al., 2008; Marcotti et al., 2010). When the drug enters the lymphatic space of the inner ear, the high-frequency region of the cochlea is the first to be damaged (Kroese et al., 1989).

Aminoglycoside antibiotics mainly accumulate in the mitochondria and lysosomes in cochlear HCs, and the increase in intracellular oxygen free radicals caused by aminoglycosides is one of the important factors leading to HC damage (Coffin et al., 2013; Liu et al., 2016, 2018; Wang et al., 2016; He et al., 2017; Waqas et al., 2017; Yu et al., 2017; Li A. et al., 2018; Li H. et al., 2018). Activated free radicals cause oxidative damage to lipids, proteins, DNA, and RNA in cells, leading to disruptions in physiological functions and to morphological defects (Cruz et al., 2007; Ferber et al., 2012). A large number of studies have shown that ototoxic drug-induced deafness, noise-induced deafness, ischemia-reperfusion-induced deafness, and presbycusis are all closely related to the increase of oxygen free radicals in HCs and subsequent apoptosis (Seidman et al., 2000; Sena and Chandel, 2012). Aminoglycosides also cause large amounts of calcium ions to enter the HCs after disruption of the mitochondrial electron transport chain, and this results in calcium overload and the release of cytochrome C into the cytoplasm that further increases mitochondrial permeability and initiates the caspase-independent apoptotic pathway (Hirose et al., 1999).

Fasudil is a novel isoquinoline sulfonamide derivative and is a calcium antagonist that effectively inhibits ROCK-II, PKA, PKG, PKC, and MLCK (Zhang and Wu, 2017). Fasudil is an FDA-approved drug and is widely used in clinical treatment because of its safety and low side effects (Mong and Wang, 2009; Liu et al., 2014). Fasudil has significant neuroprotective and therapeutic effects in the treatment of ischemic cerebrovascular disease (Rikitake et al., 2005), and it can reduce the number of macrophages and neutrophils in alveolar lavage fluid by inhibiting the Rho/Rock signaling pathway (Galiè et al., 2005; Doe et al., 2009; Yasuda et al., 2011; Jiang et al., 2012). Inhibition of the Rho/Rock signaling pathway can reduce oxidative stress and increase sulfur dioxygenase activity (Lee et al., 2017). Rho-kinase (ROCK) has been reported to be involved in many cellular functions such as cell contraction, adhesion, and migration (Ohsawa et al., 2016). Because Rho protein is mainly concentrated on the mitochondrial membrane it is thought to be involved in mitochondrial function (Orlando et al., 2006; Shen et al., 2015). In addition, the ROCK inhibitors can effectively prevent paraquat-induced cell death by reducing ROS and recovering mitochondrial membrane potential (Shen et al., 2015). However, the role of fasudil in protecting against aminoglycoside-induced HC loss has not been investigated in the inner ear.

To explore the effects of fasudil in protecting against neomycin-induced HC damage, we used cochlear whole-organ explant cultures and auditory HEI-OC-1 cells *in vitro* along with the *in vivo* neomycin damage model. We found that fasudil significantly reduced neomycin-induced HC loss both *in vitro* and *in vivo*, as well as prevented neomycin-induced hearing loss *in vivo*. We show that fasudil could significantly inhibit the Rho signaling pathway in both whole-organ cultured cochleae and HEI-OC1 cells after neomycin exposure, and this led to reduced levels of ROS accumulation and reduced apoptosis of HCs and HEI-OC1 cells. Thus, fasudil might contribute to reducing both HC loss and HEI-OC1 cell death after neomycin exposure by inhibiting the Rho signaling pathway. Taken together, our results suggest that fasudil might be a new therapeutic drug for the prevention of aminoglycoside-induced HC damage.

## Materials and Methods

### Animal Experiments

In this study, P3 mice were used for whole organ cochlear explant culture experiments, and P8 mice were used for *in vivo* experiments. WT mice were injected with 200 mg/kg neomycin daily from P8 to P14, while fasudil was injected from P6 at a dose of 2 mg/kg, which was 2 days before neomycin injection, and was continued to be injected daily for 9 days until P14. All experimental procedures were carried out in accordance with the policies of the Committee on Animal Research of Southeast University, and all efforts were made to minimize the number of mice used and their suffering. In all explant culture experiments, each group in a single experiment had no fewer than three cochlear explants, and each experiment was repeated at least four times. In the *in vivo* experiments, each experiment group had at least three mice, and each experiment was repeated at least four times.

### Cell Culture

HEI-OC-1 cells were cultured in DMEM supplemented with 10% FBS and 100 IU/ml penicillin (CSPC, H20033291). The cells were grown at 33°C with 10% CO_2_ and subcultured at 80% confluence using 0.25% trypsin/EDTA (Invitrogen; #25200-056). Neomycin (sigma, N6386-5G) was used at a final concentration of 5 mM to damage the HEI-OC-1 cells, and fasudil (Macklin, C10093618) was used at a final concentration of 0.01 μM to treat the HEI-OC-1 cells.

### Whole Organ Explant Culture

Neonatal (P3) mice were sacrificed by cervical dislocation and soaked in 75% alcohol, and the inner ear tissues including the cochlea were dissected using scissors and placed in pre-cooled sterile HBSS. The volute was opened under a microscope, and the spiral ganglion and vascular streaks were removed. The cochlea was then placed face up in a four-well dish that had been previously covered with RatCol (Advanced BioMatrix, 5153). Finally, DMEM-F12 medium containing 10% FBS was added, and the cochleae were cultured in an incubator. After culturing for 12 h, fasudil (0.01 μM) was added for 12 h. The cochleae were then co-treated with neomycin (0.5 mM) for another 12 h. After fasudil and neomycin were removed, the tissues were cultured for an additional 24 h. The number of explants in each treatment group was not less than three, and each experiment was repeated more than four times.

### *In vivo* Neomycin Damage Mouse Model

P6 mice were divided into three groups: (i) injection with normal saline from P8 until P30 with saline; (ii) subcutaneous injection with neomycin (200 mg/kg/day) from P8 to P14; and (iii) intraperitoneal injection with fasudil (2 mg/kg/day) from P6 to P7 and subcutaneous injection of neomycin from P8 to P14. The number of mice in each treatment group was not less than three, and each experiment was repeated more than four times.

### Auditory Brainstem Response (ABR) Test

Auditory brainstem response (ABR) analysis was performed in anesthetized mice at P30 to measure the hearing threshold. The hearing threshold was assessed at six frequencies (4, 8, 12, 16, 24, and 32 kHz) using a TDT system 3 (Tucker-Davies Technologies, Gainesville, FL, USA).

### Real-Time PCR

Total RNA was extracted from HEI-OC1 cells or whole-explant cultured mouse cochleae with ExTrizol Reagent (Life Technologies) and reverse transcribed to cDNA using cDNA synthesis kits (TAKARA, 6210A) according to the manufacturers’ protocols. The qRT-PCR was performed on a LightCycler 480 RT-PCR system (MJ Research) with the LightCycler 480 SYBR Green I Master Mix (Applied Biosystems). The following primers were designed for each targeted mRNA: c-jun, sense 5′-GCG TGA GTAA ACG AAA GCT GG-3′ and antisense 5′-GGT TCA AGG TCA TGC TCT GTT T-33′ ROCK1, sense 5′-GAC TGG GGA CAG TTT TGA GAC-3′ and antisense 5′-GGG CATC CAAT CCA TCC AGC-3′ aJNK, sense 5′-GTG GGG TAT GCC CAA GAG G-3′ and antisense 5′-GCC ATA AAG CCC AGA TAG AGC-3 and C-FOS, sense 5′-AAC AGA TCC GAG CAG CTT CTA-3 and C-FOS, sense 5′-AAC TGA GCT TCA ACC GGC ATC-3 and Caspase-3, sense 5′-ATG GAG AAC AAC AAA ACC TCA GT-3′ and antisense 5′-TTG CTC CCA TGT ATG GTC TTT AC-3′; Caspase-8, sense 5′-ATG GCG GAA CTG TGT GAC TCG-3′ and antisense 5′-GTC ACC GTG GGA TAG GAT ACA GCA-3′ aCaspase-9, sense 5′-CCT AGT GAG CGA GCT GCA AG-3′ and antisense 5′-ACC GCT TTG CAA GAG TGA AG-3′ aCaspase-12, sense 5′-AGA CAG AGT TAA TGC AGT TTG CT-3′ and antisense 5′-TTC ACC CCA CAG ATT CCT TCC-3′; Bax, sense 5′-TTC ATC CAG GAT CGA GCA GG-3′ and antisense 5′-CGT CAG CAA TCA TCC TCT GC-3′ aBcl-2, sense 5′-GCC TTC TTT GAG TTC GGT GG-3′ and antisense 5′-GCT GGG GCC ATA TAG TTC CA-3′ aGAPDH, sense 5′-AGG TCG GTG TGA ACG GAT TTG-3′and antisense 5′-TGT AGA CCA TGT AGT TGA GGT CA-3′. qPCR conditions consisted of an initial denaturing step of 5 min at 95°C followed by 45 cycles of 10 s denaturation at 95°C, 20 s annealing at 60°C, and 20 s extension at 72°C. The mRNA expression was normalized to the mRNA expression of *GAPDH*, and the results were calculated using the comparative cycle threshold (ΔΔCt) method.

### Flow Cytometry

MitoTracker Red CMXRos (YEASEN, 40741ES50) and DCFH-DA (Beyotime, s0033) were used to determine the mitochondrial membrane potential and to analyze mitochondrial ROS production, respectively. After trypsinization, HEI-OC1 cells were collected by centrifugation and washed with cold 1× PBS. The cell mass was resuspended in a solution containing MitoTracker Red CMXRos or DCFH-DA for 15 min at room temperature in the dark, followed by washing with cold PBS and analysis by flow cytometry (BD FACSCalibur).

FITC Annexin V (BD, 556547) was used for the analysis of apoptosis, and propidium iodide (PI) was used to distinguish viable cells from nonviable cells. HEI-OC1 cells were trypsinized for 5 min and collected by centrifugation at 1,000 rpm for 5 min, washed with cold 1× PBS, resuspended in 1× binding buffer, and aliquoted at 1 × 10^5^ cells (100 μl) into a 5 ml culture tube. FITC Annexin V and PI were added to the tube, gently vortexed, incubated for 15 min at room temperature in the dark, and analyzed by flow cytometry within 1 h.

### Western Blot

HEI-OC1 cells and cochleae were lysed with cold RIPA Lysis Buffer plus PMSF. A BCA Protein Quantification Kit (Thermo, #NCI3227CH) was used to measure the protein concentration with GAPDH as the reference protein in line with the manufacturer’s instructions. Cleaved-CASP3 was measured using an anti-cleaved-CASP3 antibody (CST, 9664S), cleaved-CASP9 was measured using an anti-Cleaved-CASP9 antibody (Abcam, ab202068), and GAPDH was measured using an anti-GAPDH rabbit polyclonal antibody (Google). Peroxidase-conjugated goat anti-rabbit immunoglobulin G (Santa Cruz Biotechnology) was used as the secondary antibody. The proteins were bound to polyvinylidene fluoride membranes, and a SuperSignal West Dura chemiluminescent substrate kit was used to detect the complexes according to the manufacturer’s instructions. The western blots were semi-quantified using ImageJ to measure the intensities of the target bands, and the ratio of the target protein to the control protein in each sample was calculated.

### Immunofluorescence

Immunohistochemistry was performed using the Fast ImmunoCytoChemistry^®^ Staining Kit (Protein Biotechnologies). Anti-cleaved-CASP3 antibody (CST, #9661), anti-Myo7a antibody (Proteus Bioscience, #:25-6790), and DAPI were used for staining apoptotic cells, staining HCs, and staining nuclei, respectively.

A TUNEL Kit (Roche, 11684817910) was used to detect apoptotic cells following the manufacturer’s instructions, and DCFH-DA was used to measure ROS levels in live cells according to the manufacturer’s instructions. For other antibody staining, the samples were incubated with 4% polyoxymethylene (Sigma, 158127) for 1 h and washed completely three times with PBST [1× PBS with 0.1% Triton X-100 (Solarbio, 1109F0521)] and permeabilized with 0.5% Triton X-100 (blocking medium) for 1 h. The primary antibodies were then added and incubated for 10 h at 4°C at a dilution of 1:400–1:1,000. The samples were washed three times with PBST and incubated for 1 h at 37°C with secondary antibody or DAPI. The samples were imaged with a confocal microscope.

### Cell Number Analysis

After incubating HEI-OC1 cells for 24 h in 96-well plates at 2,000 cells/well with three replicates, different drugs were added (controls received a similar volume of DMEM). Cell numbers were counted with the CCK-8 Cell Counting Kit (CC201, Protein Biotechnology) at different time points after incubation.

### Statistical Analysis

All data are shown as the mean ± SD, and all experiments were repeated at least three times. All statistical analyses were conducted using Microsoft Excel and GraphPad Prism 7. Two-tailed, unpaired Student’s *t*-tests were used to determine statistical significance when comparing two groups, and one-way ANOVA followed by a Dunnett multiple comparisons test was used when comparing more than two groups. A value of *p* < 0.05 was considered statistically significant.

## Results

### The Survival of HEI-OC-1 Cells Is Affected by Fasudil Concentration and Treatment Time

**Figure 1:**
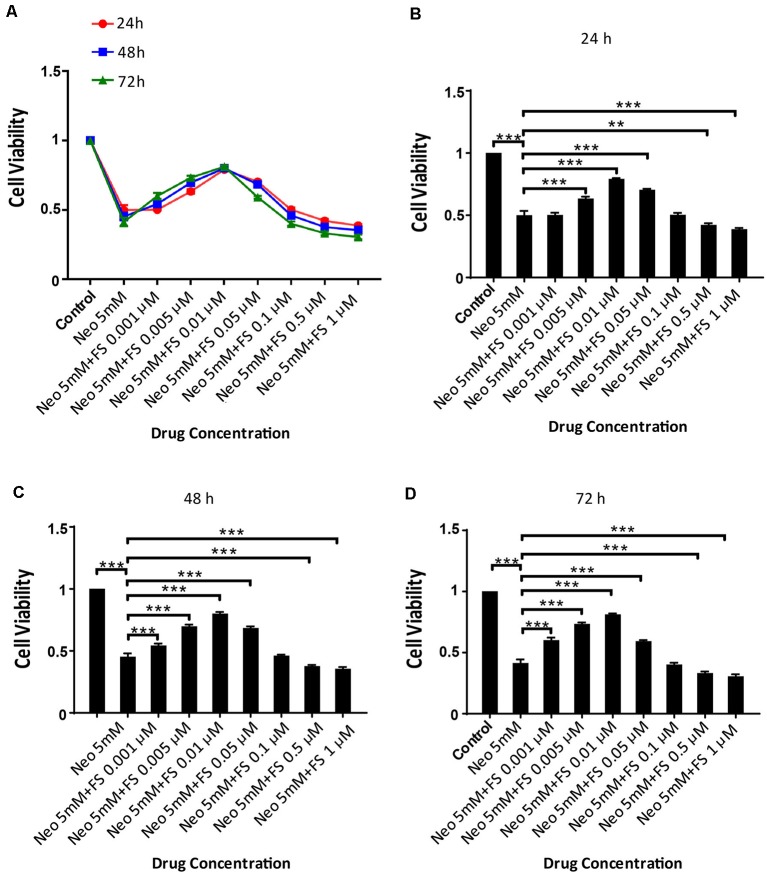
Fasudil promoted HEI-OC1 cells survival after neomycin exposure. **(A)** The number of live cells after neomycin exposure was measured with the CCK-8 kit after pretreatment with different fasudil concentrations (0.001 μM, 0.005 μM, 0.01 μM, 0.05 μM, 0.1 μM, 0.5 μM, and 1 μM) for different times (24 h, 48 h, and 72 h). **(B)** CCK-8 result of HEI-OC1 cells pre-treated with different fasudil concentrations for 24 h after neomycin exposure. **(C)** CCK-8 result of HEI-OC1 cells pre-treated with different fasudil concentrations for 48 h after neomycin exposure. **(D)** CCK-8 result of HEI-OC1 cells pre-treated with different fasudil concentrations for 72 h after neomycin exposure. **p < 0.01, ***p < 0.001.

Because there are no reports of using fasudil to protect against ototoxic drug-induced HC loss, we first investigated the appropriate dose and treatment time of fasudil in the auditory cell line HEI-OC-1 before neomycin exposure. Based on the analysis of the degree of damage to HEI-OC-1 cells by different concentrations of neomycin in our previous studies (Guan et al., 2016; He et al., 2016, 2017; Yu et al., 2017), we chose 5 mM neomycin to induce HEI-OC-1 cell damage. We pre-treated the HEI-OC-1 cells with different concentrations of fasudil (0.001 μM, 0.005 μM, 0.01 μM, 0.05 μM, 0.1 μM, 0.5 μM, and 1 μM) for different times (24 h, 48 h, and 72 h) and then treated the cells with 5 mM neomycin together with the fasudil (the same concentration as pre-treatment) for 24 h. The CCK8 results showed that the number of surviving HEI-OC-1 cells gradually increased with low concentrations of fasudil, but once the concentration of fasudil was higher than 0.01 μM the number of surviving HEI-OC-1 cells began to decrease. We also found that there was no significant difference in cell viability with the different pretreatment durations (*p* > 0.05, *n* = 3). Therefore, we chose 0.01 μM fasudil pretreatment for 24 h as the optimum treatment condition in this study (Figures 1A–D).

### Fasudil Successfully Prevents the Neomycin-Induced Cochlear HC Loss in Whole-Organ Explant Cultures *in vitro*

We next investigated the protective effects of fasudil in whole-organ explant cultures. Based on the analysis of the degree of damage to cultured cochlea by different concentration of neomycin in our previous studies (Guan et al., 2016; He et al., 2016, 2017; Yu et al., 2017), we chose 0.5 mM neomycin to induce cochlear HC damage. The cochlear explants were pre-treated with 0.01 μM fasudil for 12 h and then with 0.5 mM neomycin for another 12 h, and this was followed by immunofluorescence staining with antibodies against Myosin7a.

**Figure 2:**
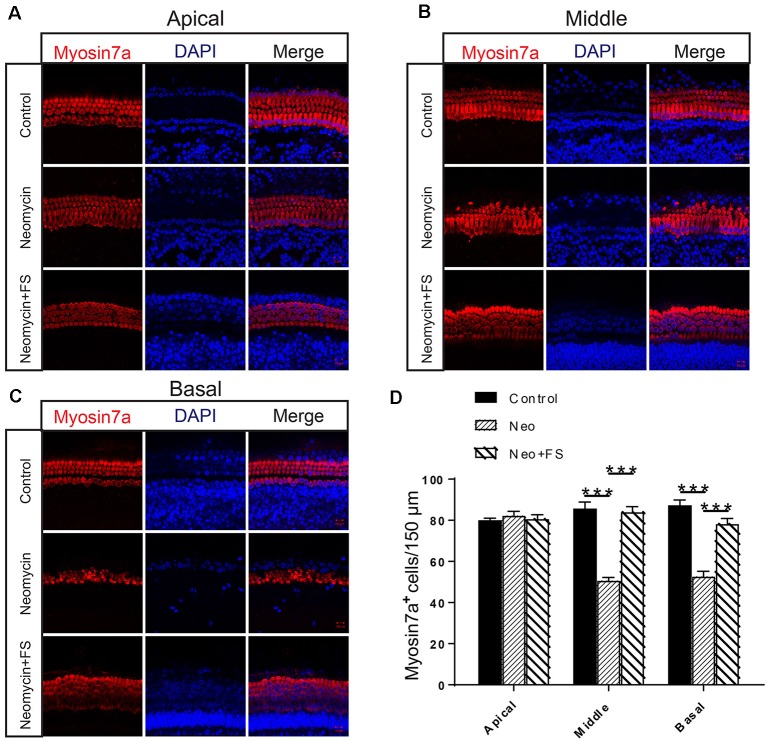
Fasudil promoted hair cell (HC) survival in the cochlea after neomycin exposure. **(A)** Immunofluorescence staining with the anti-Myo7a antibody in the apical turn of the cochlea after different treatments. **(B)** Immunofluorescence staining with the anti-Myo7a antibody in the middle turn of the cochlea after different treatments. **(C)** Immunofluorescence staining with the anti-Myo7a antibody in the basal turn of the cochlea after different treatments. **(D)** Quantification of the number of Myo7a-positive cells per 150 μM of the cochlea in **(A–C)**. ***p < 0.001.

We found that the numbers of HCs in the apical turn of the cochlea showed no significant differences after neomycin exposure with or without fasudil treatment compared to the undamaged control group (Figures 2A,D, *p* > 0.05, *n* = 3). In contrast, the HC numbers in the middle and basal turns of the cochlea were significantly decreased after neomycin treatment (Figures 2B–D, *p* < 0.001, *n* = 3), and HC loss was successfully prevented by treatment with fasudil in the middle and basal turns (Figures 2B–D, *p* < 0.001, *n* = 3).

### Fasudil Successfully Reduces Neomycin-Induced Cochlear HC Loss and Hearing Loss *in vivo*

**Figure 3:**
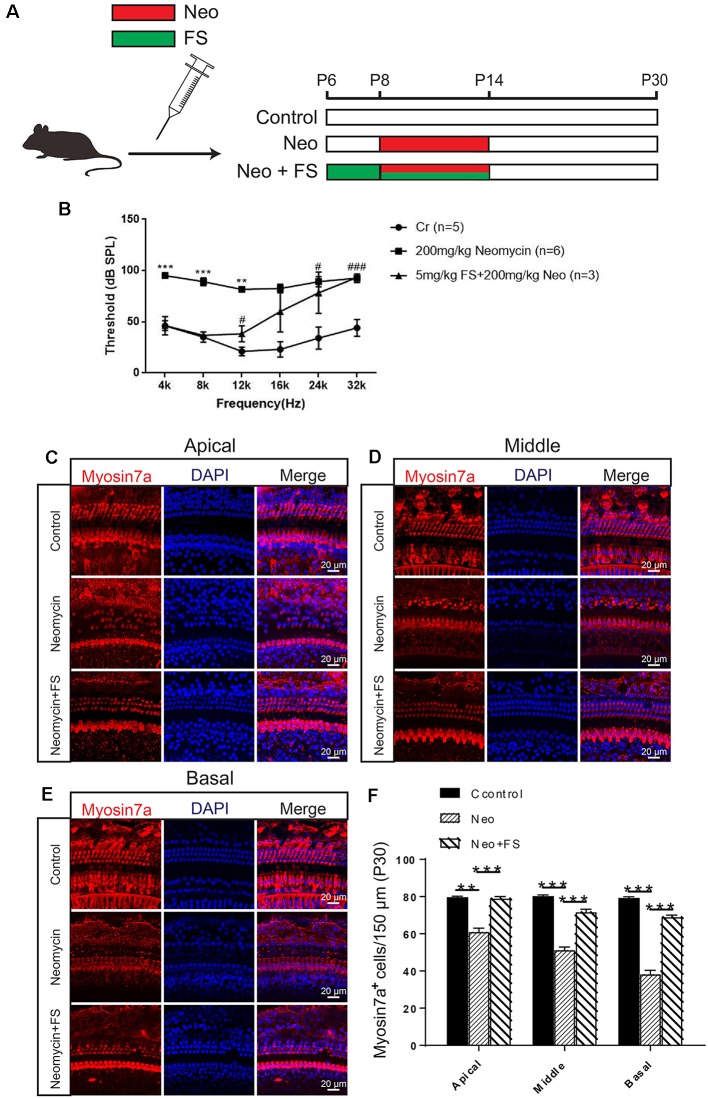
Fasudil promoted HC survival in the cochlea after neomycin exposure. **(A)** The three different treatment groups. Control mice were injected with normal saline from P8 until P30, mice in the neomycin group were subcutaneously injected with neomycin (200 mg/kg/day) from P8 to P14, and mice in the fasudil treatment group were intraperitoneally injected with fasudil (2 mg/kg/day) from P6 to P7 and co-treated with neomycin from P8 to P14. **(B)** The results showed that the hearing threshold of P30 mice increased after neomycin treatment and was reduced by fasudil. **(C)** Immunofluorescence staining with the anti-Myo7a antibody in the apical turn of the cochlea after different treatments. **(D)** Immunofluorescence staining with the anti-Myo7a antibody in the middle turn of the cochlea after different treatments. **(E)** Immunofluorescence staining with the anti-Myo7a antibody in the basal turn of the cochlea after different treatments. **(F)** Quantification of the number of Myo7a-positive cells per 150 μM of the cochlea in **(A–C)**. *represents Control vs 200 mg/kg Neomycin, ^#^represents Control vs 5 mg/kg FS + 200 mg/kg Neomycin, **p < 0.01, ***p < 0.001, ^#^*p* < 0.05, ^###^*p* < 0.001.

In order to confirm the protective effects of fasudil after neomycin treatment *in vivo*, we took advantage of the *in vivo* neomycin damage model as previously reported (Choi et al., 2014; Sun et al., 2014). WT mice were injected with 200 mg/kg neomycin daily from P8 to P14, while fasudil was injected from P6 at a dose of 2 mg/kg, which was 2 days before neomycin injection, and was continued to be injected daily for 9 days until P14 (Figure 3A). We then performed the ABR test to measure the hearing threshold of the mice at P30 (Figure 3B). We found that the hearing thresholds were increased at all frequencies in the neomycin-only group compared with the undamaged controls. In the fasudil treatment group, the hearing loss was significantly reduced at low frequency and middle frequency (4 kHz, 8 kHz, 12 kHz, and 16 kHz) compared to the neomycin-only group (*p* < 0.001, *n* = 3), while at high frequency (24 kHz and 32 kHz) the protective effects were not significant (Figure 3B). In addition, immunofluorescence staining with antibodies against Myosin7a showed that the HC number was significantly decreased in all three turns of the cochlea after neomycin treatment compared to the undamaged control (Figures 3C–F, *p* < 0.01, *n* = 3). In addition, treatment with fasudil significantly increased the surviving HC number in all three turns compared to the neomycin-only group, suggesting that fasudil treatment successfully protected against neomycin-induced HC loss in all three turns *in vivo* (Figures 3C–F, *p* < 0.001, *n* = 3).

### Fasudil Successfully Prevents Neomycin-Induced Cell Death and Apoptosis in Auditory HEI-OC-1 Cells

**Figure 4:**
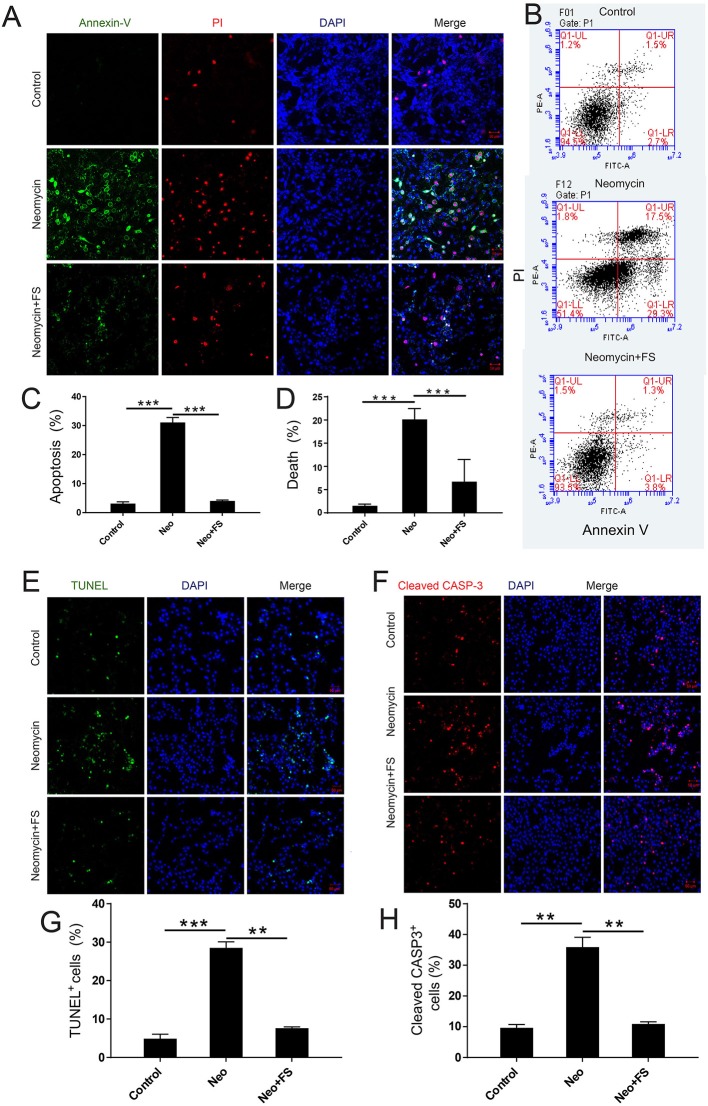
The effect of fasudil (FS) on neomycin-induced HEI-OC1 cell apoptosis. **(A)** Images of HEI-OC1 cells stained with Annexin V (green) and propidium iodide (PI, red). **(B)** Apoptosis analysis by flow cytometry after different treatments. **(C,D)** The proportions of early apoptotic and death cells in **(B)**. **(E)** TUNEL and DAPI double staining showing the apoptotic HEI-OC1 cells after different treatments. **(F)** Cleaved-CASP3 and DAPI double staining showing the apoptotic HEI-OC1 cells after different treatments. **(G)** Quantification of the numbers of TUNEL/DAPI double-positive cells in **(D)**. **(H)** Quantification of the numbers of TUNEL/DAPI double-positive cells in **(E)**. **p < 0.01, ***p < 0.001.

To investigate the protective effects of fasudil in auditory HEI-OC-1 cells, we used PI to label the dead cells and Annexin V to label the cells undergoing apoptosis. Annexin V recognizes and binds to phosphatidylserine, which is transferred from the intracellular leaflet to the external leaflet of the plasma membrane in apoptotic cells, while the DNA-binding dye molecule PI can only enter cells when their membranes are ruptured. The HEI-OC1 cells were pre-treated with 0.01 μM fasudil for 24 h and then treated with 5 mM neomycin together with 0.01 μM fasudil for another 24 h. We found that the proportions of apoptotic cells and dead cells were both significantly increased in the neomycin-only group compared to the undamaged controls (Figures 4A–D, *p* < 0.001, *n* = 3), while fasudil treatment prevented neomycin-induced cell apoptosis and cell death (Figures 4A–D, *p* < 0.001, *n* = 3). The proportions of apoptotic cells and dead cells were both very similar to undamaged controls after treatment with fasudil (Figures 4A–D).

**Figure 5:**
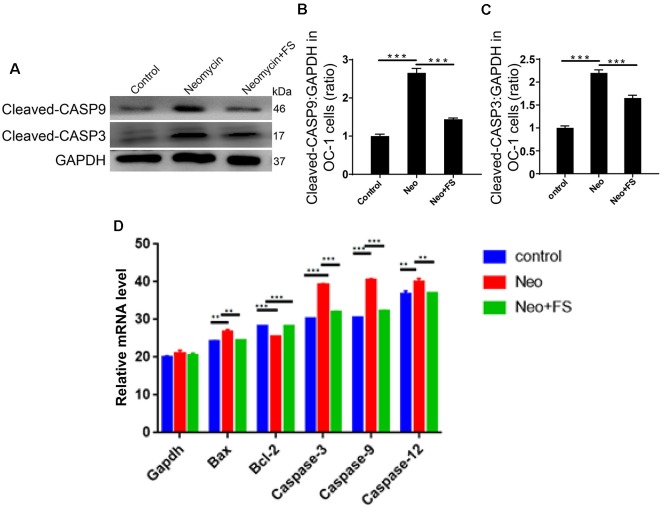
Changes in apoptosis-related protein and gene expression after neomycin and fasudil (FS) treatment. **(A)** Western blots with anti-cleaved-CASP3 antibody and anti-cleaved-CASP9 antibody showed that the neomycin-induced increase in cleaved-CASP3 and cleaved-CASP9 was largely attenuated by pretreatment with fasudil. **(B,C)** Quantification of the western blot in **(A)**. **(D)** The mRNA levels of pro-apoptotic and anti-apoptotic genes were analyzed by qPCR. **p < 0.01, ***p < 0.001.

We also used caspase-3 and TUNEL staining to evaluate apoptosis in HEI-OC-1 cells and found that the numbers of caspase-3-positive and TUNEL-positive cells in the neomycin-only group were significantly greater than the undamaged controls (Figures 4E–H, *p* < 0.001, *n* = 3). Fasudil treatment successfully rescued the neomycin-induced cell apoptosis, and the proportions of caspase-3-positive and TUNEL-positive cells were similar to the undamaged controls after treatment with fasudil (Figures 4E–H, *p* < 0.001, *n* = 3). Western blot also showed that the protein expression levels of cleaved-CASP9 and cleaved-CASP3 in the neomycin-only group were both significantly increased compared to the undamaged controls (Figures 5A–C, *p* < 0.001, *n* = 3), while fasudil treatment successfully rescued the neomycin-induced increase of cleaved-CASP9 and cleaved-CASP3 (Figures 5A–C, *p* < 0.001, *n* = 3). We also performed a qPCR (quantitative real-time PCR) experiment to verify the expression of apoptosis-related genes in HEI-OC1 cells after fasudil treatment and neomycin exposure. The expression levels of the pro-apoptotic marker genes *Bax*, *Casp12*, *Casp9*, and *Casp3* were significantly higher in the neomycin-only group compared with the undamaged controls (Figure 5D, *p* < 0.01, *n* = 3), while the expression of *Bcl2*, which is an anti-apoptotic gene, was decreased in the neomycin-only group compared to the controls (Figure 5D, *p* < 0.01, *n* = 3). After fasudil treatment, the expression levels of both the pro-apoptotic genes and the anti-apoptotic gene were similar to the undamaged control group (Figure 5D, *p* < 0.01, *n* = 3). Together, these results showed that fasudil treatment significantly protected against neomycin-induced cell apoptosis and cell death in HEI-OC-1 cells.

### Fasudil Rescues the Cell Morphology of HEI-OC-1 Cells After Neomycin Exposure

In this experiment, HEI-OC-1 cells were treated with 0.01 μM fasudil for 24 h and then with 5 mM neomycin together with 0.01 μM fasudil for another 24 h. Immunofluorescence staining with antibodies against the HC marker Myosin7a and with the cell nucleus marker DAPI showed that the nuclear area was smaller and that the cellular morphology was altered after neomycin exposure compared to untreated controls (Supplementary Figures S1A,B). Fasudil treatment rescued the cell morphology of HEI-OC-1 cells after neomycin exposure, and the nuclear area and morphology of HEI-OC-1 cells were similar to the untreated control group after fasudil treatment (Supplementary Figures S1A,B).

### Fasudil Attenuates Neomycin-Induced Oxidative Stress in HEI-OC1 Cells

**Figure 6:**
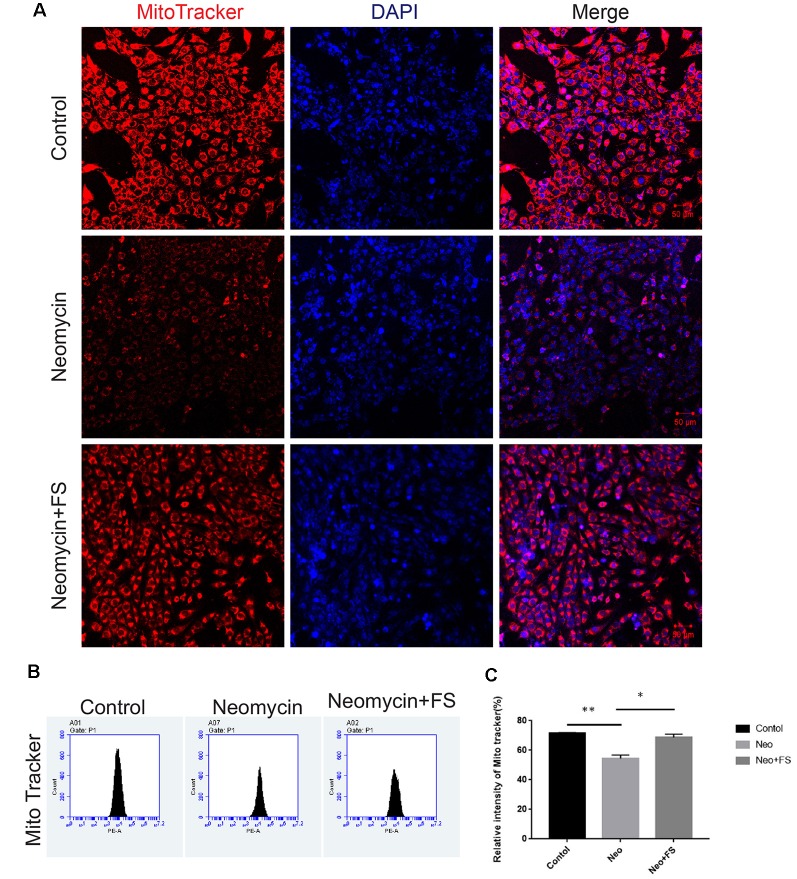
Fasudil maintains the mitochondrial membrane potential after neomycin exposure. **(A)** HEI-OC-1 cells were labeled using the MitoTracker Red CMXRos staining kit. **(B)** The analysis of mitochondrial membrane potential by flow cytometry. **(C)** Quantification of the data in **(B)**. *p < 0.05, **p < 0.01.

The production of ROS by the mitochondria has been reported to be a major factor in aminoglycoside-induced HC damage by stimulating apoptosis in the cochlea (Huang et al., 2000; Balaban et al., 2005). Here, we investigated mitochondrial function and the level of oxidative stress after fasudil treatment and neomycin exposure. HEI-OC-1 cells were treated with 0.01 μM fasudil for 24 h and then with 5 mM neomycin together with 0.01 μM fasudil for another 24 h. We used the MitoTracker Red CMXRos kit to evaluate the changes in mitochondrial membrane potential (Samudio et al., 2005). Immunohistochemistry and flow cytometry data both showed that MitoTracker intensity was significantly decreased in the neomycin-only group compared with the undamaged controls (Figures 6A–C, *p* < 0.01, *n* = 3), while fasudil treatment successfully rescued the mitochondrial membrane potential of HEI-OC-1 cells (Figures 6A–C, *p* < 0.05, *n* = 3). The MitoTracker intensity was similar to the undamaged controls after fasudil treatment. These results indicated that fasudil can prevent neomycin-induced mitochondrial dysfunction in HEI-OC-1 cells.

**Figure 7:**
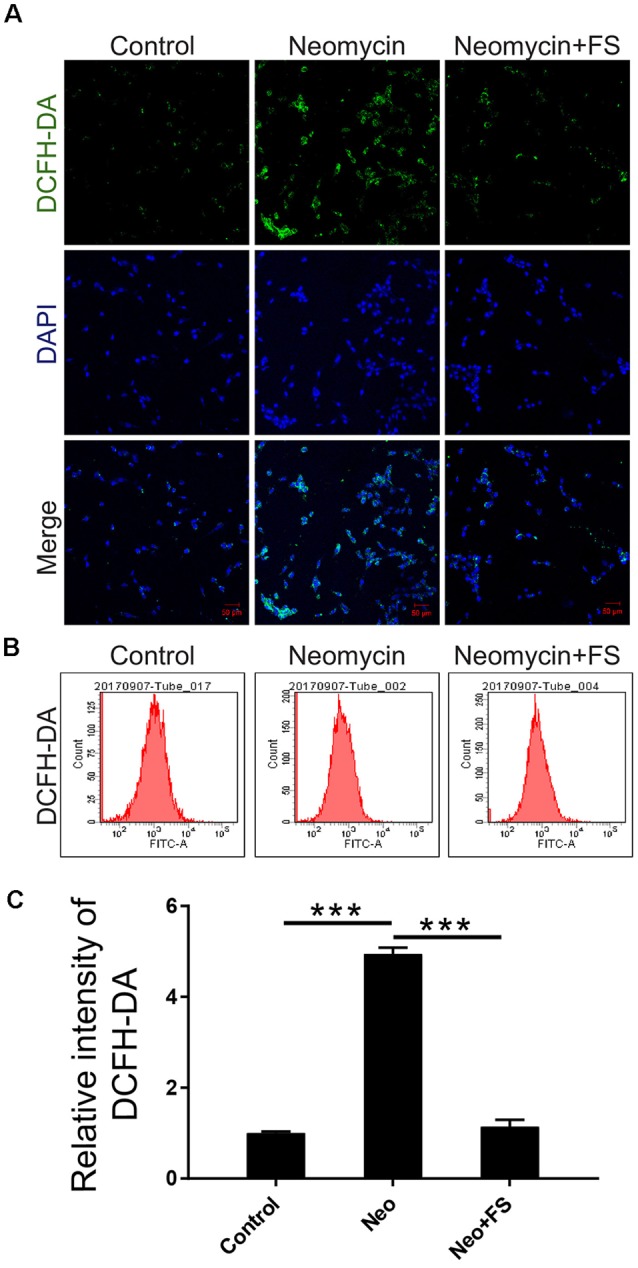
The changes in ROS levels in HEI-OC-1 cells after neomycin and fasudil (FS) treatment. **(A)** Three different groups of HEI-OC-1 cells were labeled using the DCFH-DA staining kit. **(B)** Flow cytometry data confirmed the results in **(A)**. **(C)** Quantification of the data in **(B)**. ***p < 0.001.

Next, we used DCFH-DA, which is a fluorimetric probe that selectively detects oxidative species (Aranda et al., 2013), to evaluate the ROS levels in HEI-OC-1 cells after fasudil treatment and neomycin injury. Immunohistochemistry and flow cytometry results showed that the ROS levels were significantly increased in the neomycin-only group compared to the undamaged controls (Figures 7A–C, *p* < 0.001, *n* = 3), while fasudil treatment successfully reduced the ROS levels to similar levels as in the undamaged controls (Figures 7A–C, *p* < 0.001, *n* = 3).

### Fasudil Significantly Inhibits the Rho Signaling Pathway in HEI-OC1 Cells

**Figure 8:**
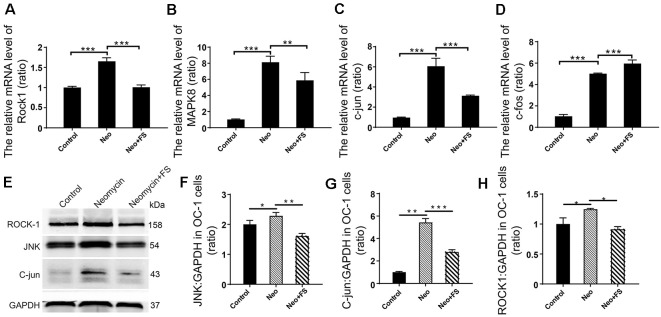
Differential expression analysis of Rho signaling pathway-related genes in HEI-OC1 cell after different treatment. **(A–D)** The mRNA levels of *Rock1*, *Mapk8*, *c-jun*, and *c-fos*. The values of each group are relative values, and the value of controls is set to 1. **(E)** The protein levels of JNK, ROCK1, and c-jun. **(F–H)** Quantification of the western blot in **(E)**. Analysis of the ratio of the target protein to the control protein in each sample. *p < 0.05, **p < 0.01, ***p < 0.001.

Previous studies reported that the neuroprotective effects of fasudil are through inhibition of the Rho signaling pathway (Mong and Wang, 2009; Liu et al., 2014). To further determine the HC protective mechanism of fasudil after neomycin exposure, we also investigated the activation of the Rho signaling pathway after fasudil treatment and neomycin exposure in auditory HEI-OC1 cells. We measured the expression levels of several key factors of the Rho signaling pathway, including ROCK1, MAPK, c-jun, JNK, and c-fos. The qPCR results showed that the expression of *Rock1*, *Mapk8*, *c-jun*, and *c-fos* was significantly increased in HEI-OC1 cells after neomycin exposure (Figures 8A–D, *p* < 0.001, *n* = 3), while after fasudil treatment the expression of *Rock1*, *Mapk8*, and *c-jun* was significantly down-regulated and the expression of *c-fos* was significantly up-regulated compared to the neomycin-only group (Figures 8A–D, *p* < 0.01, *n* = 3). Consistent with this, the western blot results also showed that the levels of JNK, c-jun, and ROCK1 were significantly increased when cells were treated with neomycin (Figures 8E–H, *p* < 0.05, *n* = 3), while fasudil treatment significantly reduced the expression of JNK, c-jun, and ROCK1 compared to the neomycin-only group (Figures 8E–H, *p* < 0.05, *n* = 3).

## Discussion

Aminoglycoside antibiotics can have ototoxic side effects, and this limits their clinical application (Durante-Mangoni et al., 2009; Zimmerman and Lahav, 2013). Aminoglycosides can induce the production of a large number of highly toxic ROS, and these superoxides are mainly concentrated in the auditory HC region, which is the main target of aminoglycoside damage (Nadol, 1993). The cochlea is a metabolically active tissue that produces ROS, and the metabolic activity in the basal turn of the cochlea is greater than that in the apical turn. Therefore, more free radicals are generated in the basal turn than in the apical turn, and the basal turn is, therefore, the most susceptible to free radical damage (Kwon et al., 2015). The ROS produced by mitochondrial metabolism under normal conditions is removed by the antioxidant mechanisms of the cells. However, aminoglycoside exposure induces large increases in ROS production in the cochlear HCs that overwhelm the cellular defense mechanisms (Chen et al., 2015; Quan et al., 2015; Esterberg et al., 2016). Therefore, finding ways to prevent aminoglycoside-induced ototoxicity by decreasing the mitochondrial production of ROS in cochlear HCs is a primary focus in the hearing research field.

Fasudil is a potent Rock inhibitor that has been used for the treatment of cerebral vasospasm and pulmonary hypertension as well as for preventing the cognitive decline seen in stroke victims and Alzheimer’s patients (Shibuya and Suzuki, 1993; Doggrell, 2005; Huentelman et al., 2009). The Rho/Rock signaling pathway is composed of key signaling molecules such as Rho GTPase, Rocks, and myosin light chain phosphatase. The Rho protein family consists of guanosine triphosphate (GTP)-binding proteins that have GTPase activity and are capable of binding and hydrolyzing GTP, allowing the protein to repeatedly circulate between the GTP-bound state (active form) and the GDP-bound state (inactive form). The different forms of Rho can further trigger downstream kinase cascades, thereby exerting multiple biological effects (Surma et al., 2011; Antoniu, 2012; Amin et al., 2013). Fasudil can also attenuate cardiac fibrosis by inhibiting the RhoA/RhoA-kinase pathway (Lee et al., 2017).

The application of fasudil in the inner ear and the related mechanisms of its activity in the inner ear have not been reported before. In this study, we explored the role of fasudil in neomycin-induced HC apoptosis in both *in vitro* and *in vivo* experiments. In our *in vivo* experiments, we found fasudil could significantly attenuate neomycin-induced hearing loss and cochlear HC damage. Although *in vitro* experiments cannot fully represent the mechanisms and phenomena as in *in vivo* experiments, *in vitro* experiments have been used by many researchers for investigation of inner-ear related mechanisms because they are convenient to perform and observe. The results of the *in vitro* experiments also showed that fasudil significantly prevented neomycin damage to cochlear HCs and auditory HEI-OC1 cells, which is consistent with the results of the *in vivo* experiments. Fasudil treatment can prevent disruption of mitochondrial function and can prevent neomycin-induced accumulation of ROS oxidative damage thus preventing apoptosis in HEI-OC1 cells and cochlear HCs after neomycin exposure. In addition, our *in vitro* studies showed that fasudil protects against neomycin-induced HC loss in the basal and middle turns of the cochlea, while the *in vivo* animal model demonstrated that the protection against hearing loss occurs in the regions corresponding to the apical and middle turns of the cochlea and that the basal part, which detects high frequencies, is not significantly protected. The results of immunofluorescence staining showed that fasudil significantly enhanced HC viability in both *in vivo* and *in vitro* experiments. However, the ABR hearing test results showed that fasudil had a limited protective effect on high-frequency hearing. This is likely because hearing capability is very sensitive, and as little as a 20% reduction in HC number can cause complete hearing loss. Thus, although fasudil significantly enhanced the survival of the HCs in the basal turn of cochlea after neomycin treatment *in vivo*, fasudil could not maintain the HC number as the same level as controls, and the number of damaged HCs was sufficient to induce significant hearing loss. Another possible reason that fasudil appeared to have a limited protective effect on high-frequency hearing in our study is that we used the HC marker Myo7a to perform the immunostaining, and it is possible that some of the HCs undergoing apoptosis still had Myo7a staining even though they no longer had normal HC function.

In summary, we provide the first report that fasudil can successfully prevent aminoglycoside-induced HC loss and hearing loss both *in vitro* and *in vivo*. We show that fasudil contributes to the increased viability of HCs after neomycin exposure by inhibiting the Rho signaling pathway, which further inhibits ROS accumulation and maintains mitochondrial function in HCs. Our findings suggest that fasudil might have clinical potential in protecting against aminoglycoside-induced HC loss and hearing loss.

## Data Availability Statement

All datasets generated for this study are included in the article/Supplementary Material.

## Ethics Statement

The animal study was reviewed and approved by Affiliated Drum Tower Hospital of Nanjing Medical University.

## Author Contributions

YZ, WL and RC conceived and designed the experiments. YZ, WL, YW and BS performed the experiments. YZ, ZH, CC, SZ, MT, XQ, WK, RC and XG analyzed the data. HW gave guidance on audiometry experiments. YZ, ZH, RC and XG wrote the article.

## Conflict of Interest

The authors declare that the research was conducted in the absence of any commercial or financial relationships that could be construed as a potential conflict of interest.
