# Supplementary material for: Pre-treatment With Fasudil Prevents Neomycin-Induced Hair Cell Damage by Reducing the Accumulation of Reactive Oxygen Species
Source: Front Mol Neurosci. 2019 Nov 6;12:264. doi: 10.3389/fnmol.2019.00264 (PMC6851027; doi:10.3389/fnmol.2019.00264)
Supplement: Supplementary file 1 [file Data_Sheet_1.PDF]

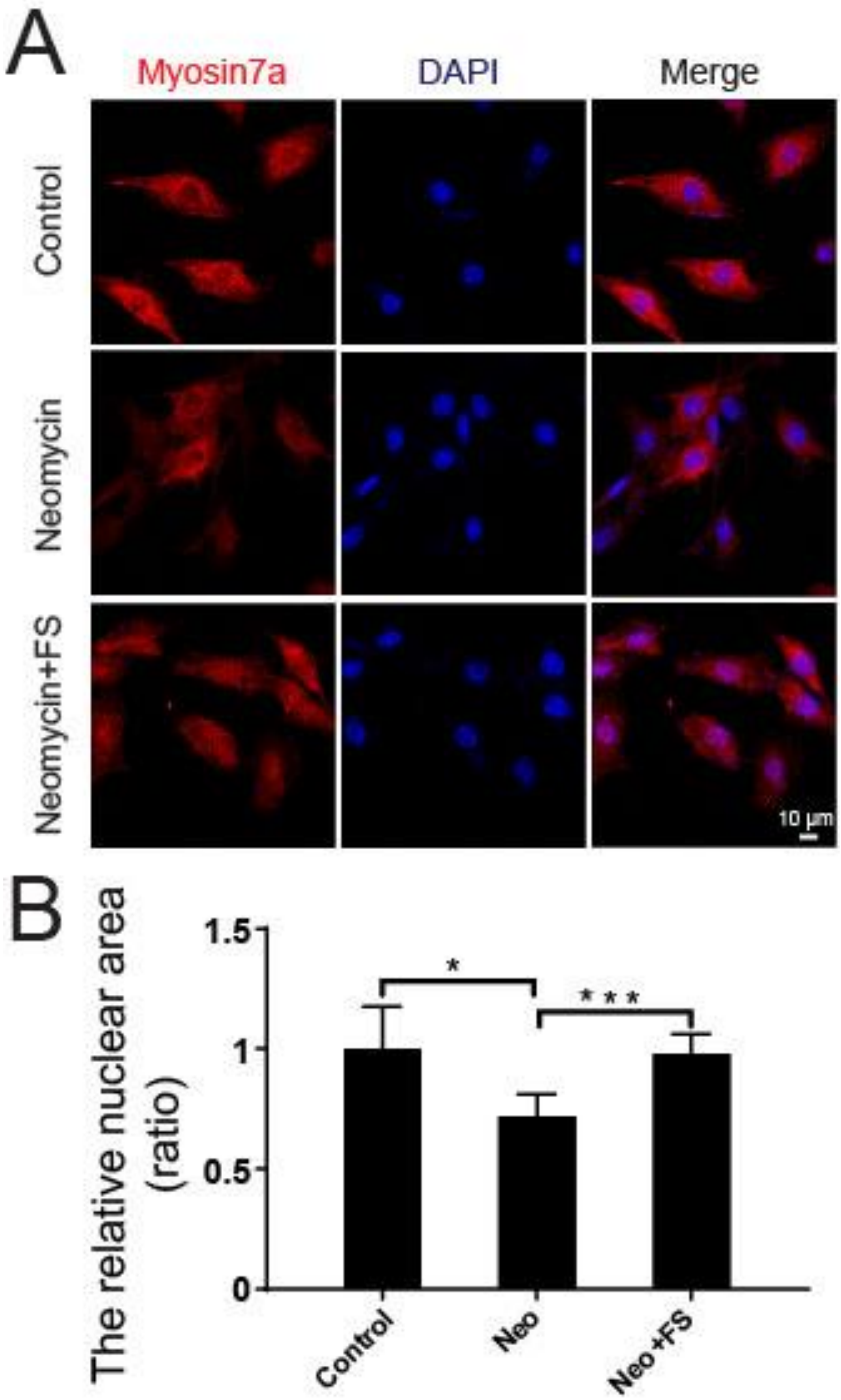

Sup-Figure 1 The changes in cell morphology after neomycin and fasudil (FS) treatment. Cell morphology in the neomycin-only group was changed, and the nuclei showed pyknosis. (A)

Images of HEI-OC1 cells stained with Myosin7a (red) and DAPI (blue). (B) Analysis of nuclear area after different treatments. * $p < 0.05$, *** $p < 0.001$.
